# “Cancer as the General Population Knows It”: Knowledge, Fear, and Lay Education in 1950s Britain

**DOI:** 10.1353/bhm.2007.0013

**Published:** 2007

**Authors:** Elizabeth Toon

**Keywords:** health education, cancer, National Health Service, Great Britain, Manchester Committee on Cancer, Ralston Paterson, “cancerphobia, ” patient experience, palliative care

## Abstract

This article examines British medical debates about cancer education in the 1950s, debates that reveal how those responsible for cancer control thought about the public and their relationship to it, and what they thought the new political economy of medicine introduced by the National Health Service would mean for that relationship. Opponents of education campaigns argued that such programs would add to the economic and organizational pressures on the NHS, by setting in motion an ill-informed, uncontrollable demand that would overwhelm the service. But an influential educational “experiment” devised by the Manchester Committee on Cancer challenged these doubts, arguing that the public’s fear was based in their experience with family and friends dying of the disease. The challenge for cancer control, then, was to improve that experience and thus change experiential knowledge.

Just after the Second World War, gynecologist Malcolm Donaldson set out to convince the British Empire Cancer Campaign (BECC) to expand its prewar program of lay cancer education. Though he chaired the BECC’s Educational Committee, Donaldson failed to persuade the organization’s [Other P-116] leaders that a national cancer-education campaign would be worthwhile. Nor would the Ministry of Health accept Donaldson’s suggestions, its medical officers arguing that “the time was not ripe for an approach to the general public.”^1^ When Donaldson managed to recruit allies in the Central Council for Health Education (CCHE), he and they soon found that neither the BECC nor the Ministry would cooperate with them on a national program. Instead, these organizations suggested that lay education about cancer should be left to the initiative of local groups choosing to experiment with it.

To scholars familiar with the history of cancer control, the BECC’s and the Ministry’s reluctance to create or participate in national educational programs about the “dread disease” might seem odd. In North America, equivalent organizations had just increased their substantial prewar commitment to education, funding national campaigns intended to “fight cancer with knowledge,” as the American Cancer Society’s motto put it.^2^ But in Britain, much of the cancer elite—the clinicians, researchers, public health workers, and government officials who made the disease their business—rejected the idea that they should teach the public about cancer symptoms and treatment. Furthermore, when cancer-education programs were developed and adopted in 1950s Britain, many differed from those in other national contexts: cancer-control organizations elsewhere used the nationally coordinated media “blitz,” the big-screen film, and the glossy color pamphlet to get their message across, while British [Other P-117] proponents of cancer education devised local efforts, often relying on the humbler media of newspapers and small-group discussions to promote everyday understanding of the disease and its treatment.

What accounts for the British cancer elite’s rejection of lay education in the immediate postwar years? And why did strategies for lay cancer education in 1950s Britain differ from those devised elsewhere? Answering these questions helps us see how those responsible for cancer control thought about the public and their relationship to it, and what they thought the new political economy of medicine introduced by the National Health Service (NHS) would mean for that relationship. In the first half of this article I explore the postwar debate over cancer education, examining why opponents of such programs objected so strongly to them. The men and women of the British cancer establishment conceived of themselves as managing the frequently irrational demands of a public they characterized as gullible and emotional, a conceptualization of “the public” that they shared with cancer experts abroad and with other medics at home.^3^ But many also believed that the public was *so* irrational about this disease that education—defined as the large-scale mass-media provision of facts about potential symptoms—was counterproductive. What was more, they argued, popular education could only add to the economic and organizational pressures on the NHS, by setting in motion an ill-informed, uncontrollable demand that would overwhelm the services they had labored to establish.

In the second half of the article I examine an influential experiment that challenged existing doubts about cancer education by offering a new model of what cancer education was and what it needed to do. The Manchester Committee on Cancer (MCC) argued that the British public’s knowledge came from local, everyday encounters with medical institutions and expertise in their communities. While these proponents of education agreed that the everyday dread of cancer was substantial and frequently irrational, they also argued that the public’s fear was understandable and based in reality: it derived from personal experience with family, friends, and neighbors dying of the disease. The real challenge for cancer control, then, was to improve that experience and thus change experiential knowledge, “cancer as the general population knows it.” To accomplish this, the Manchester organizers instituted a very different sort of educational program, one that enlisted the voices of everyday Britons and mobilized existing social networks.[Other P-118]

## Much Anxious Thought: What Was Wrong with Lay Cancer Education?

In Britain, discussions about lay cancer education began in the early twentieth century, largely spurred by Charles P. Childe’s 1906 book *The Control of a Scourge*.^4^ Childe and his allies agreed among themselves and with their international colleagues on a general model of what cancer education was, whom it would address, and what its results would be. First, like others involved in popular health instruction, advocates of cancer education believed themselves to be remedying mass ignorance through the provision of scientific facts, which would then encourage sensible health behavior. Cancer, the medical elite agreed, was the most frightening of diseases, and fear of it could drive otherwise intelligent people into irrational behavior. Cancer education thus needed to bring the everyday Briton to her or his senses—to replace an unthinking fear with a reasoned, appropriate response. Second, women formed the primary audience for cancer education. This grew in part from notions about women’s nature and roles: women were said to be more likely to be irrational in the face of cancer, but were also considered more likely to bear responsibility for monitoring their family’s health.^5^ Third, educators agreed that their focus needed to be the “accessible cancers,” those more amenable to early detection by the patient or the general practitioner. Breast and cervical cancer were the most prominent of these, meaning that women seemed to form a disproportionate portion of the population that could be helped by education.

The most powerful assumption about cancer education, though, was that its central message should be that “early cancer is curable.” This message, it was thought, would cultivate popular awareness of symptoms that might mean cancer, but would also reduce the public’s fear of cancer. If knowledge was increased and fear decreased, the argument went, everyday women and men would present themselves to their GPs as soon as they noticed anything amiss. Any cancer present would be diagnosed at an earlier stage, and its cure would be likelier. In other words, British advocates of lay cancer education endorsed the view that “delay” was their chief foe, drawing on the same discourses as their colleagues elsewhere (but especially in the United States).^6^ A few admitted cautiously that earlier diagnosis would not necessarily increase the likelihood of cure for some [Other P-119] cancers and for some individual patients, and even that earlier presentation by patients might not make much of a dent in overall mortality rates. Nonetheless, they believed that a lay educational campaign would, generally, decrease the length of time that patients “delayed” seeking advice after noticing symptoms, which would—again, generally—positively affect the stage distribution of cancers detected, and thereby positively affect cure rates. Other advocates of lay education seem not to have concerned themselves with such complicated caveats. But regardless of how many qualifications they offered to the delay argument, proponents agreed fervently that by increasing the public’s knowledge they would demolish a widespread fear sown by equally widespread ignorance.^7^

Nevertheless, much of the British medical community opposed educating laypeople about cancer, and thus relatively little lay cancer education was done in the 1910s, 1920s, and 1930s. As Ornella Moscucci has shown, early twentieth-century opponents of lay education worried that it would foster “cancerphobia,” pollute public discourse, and (by facilitating lay knowledge) undermine professional authority.^8^ Some local health authorities offered lectures about cancer to lay audiences, but when national organizations like the British Empire Cancer Campaign addressed the public, they did so mostly for fund-raising purposes. In the 1930s the BECC made a formal distinction between fund-raising appeals and education, vesting the latter responsibility in a Propaganda Committee headed by Malcolm Donaldson, a gynecological surgeon at London’s St. Bartholomew’s Hospital and a National Radium Commission member.^9^ Donaldson organized speaker panels in several counties, outfitting local practitioners with “skeleton lectures” to guide their presentations.^10^ While the talks were apparently popular and several medics cooperated by giving lectures, the Propaganda Committee was discontinued when the war began.^11^

When the war ended, Donaldson and others expected the BECC to restart its educational initiatives, but the Campaign’s leadership responded that it was giving lay cancer education “much anxious thought.”^12^ They did reconstitute Donaldson’s prewar Cancer Propaganda Committee, now renamed the Cancer Education Committee, in 1947; however, this committee was not expected to conduct educational campaigns, but to *consider* whether the BECC *should* conduct educational campaigns.^13^ Two years later, the Education Committee proposed that the BECC should expand its lectures on cancer for general practitioners, and should also begin a local “test scheme” of lay education—but the Executive Committee took two years to mull over the proposal, finally announcing that first “the views of all general practitioners throughout the country should be obtained.”^14^ So along with a booklet urging recipients to become more cancer-conscious in their practice, the BECC sent a questionnaire to all general practitioners in the United Kingdom, asking whether they thought lay education “would be of assistance in securing the earlier diagnosis of cancer, and thereby improving the chances of cure.”^15^

While the BECC surveyed the nation’s doctors, the Ministry of Health also contemplated lay cancer education, spurred by inquiries from the Central Council for Health Education. This small, quasi-official body had been set up before the war; drawing largely on financial contributions from local authorities, the CCHE produced health-education materials that local authorities could use in their own campaigns, and trained local personnel in educational methods. The CCHE had contacted both the Ministry and the BECC to ask whether they wanted to collaborate on cancer-education materials, but the BECC had declined to respond, instead waiting to see what role vis-à-vis cancer education the Ministry would suggest for the CCHE.^16^ Ministry officials expressed their annoyance at the CCHE’s push to “do something” despite other bodies’ disapproval, and argued that the Council should simply advise local authorities.^17^ Producing cancer materials or organizing educational programs, [Other P-121] the Ministry insisted, was impossible before the National Health Service was fully in place.^18^

In 1949, Ministry officials decided to consult the Central Health Service Council’s Standing Cancer and Radiotherapy Advisory Committee (SCRAC) on the matter. Citing the “good deal of propaganda” disseminated by American and Canadian cancer societies, the memorandum noted that Ministry leaders were “very doubtful whether any general approach to the public on these lines would, in this country, be desirable.”^19^ The SCRAC—composed of leading cancer clinicians and researchers advising the Ministry—debated the issue, and, as internal discussions later revealed, “[they] were, in fact, so divided that they did their best not to give any advice at all.”^20^ After a few more months the SCRAC agreed that local authorities could undertake educational programs, but implied strong disapproval of any national scheme; the Central Health Services Council endorsed this decision by its Committee. With this guidance, Ministry officials worked to dissuade the CCHE from undertaking anything that could be construed as a national cancer-education program.^21^ Finally in early 1951, after much negotiation, the SCRAC agreed it would not object to a CCHE pilot survey on cancer education, although the Ministry made it clear that no government funds would be forthcoming for such a survey.^22^ Soon afterward, the SCRAC decided that a national program of lay education was still premature, but that central government (meaning the Ministry) should encourage local authorities and voluntary bodies to explore the effectiveness of such programs.^23^

In late 1952, the Ministry conditionally approved educational “test schemes” and began drafting a circular for local health authorities, suggesting that they avail themselves of the CCHE’s model scheme.^24^ But before sending out this circular, the Ministry sent it to the British Medical Association’s General Medical Services Council (GMSC), asking its opinion on the matter. (Such consultation was common.) The GMSC, [Other P-122] misreading the circular as a Ministry endorsement of a national lay educational campaign, angrily resolved that it was “doubtful as to the wisdom of instituting a campaign of this nature,” and did not wish to be associated with such a scheme.^25^ The Ministry of Health responded rapidly, assuaging the GMSC’s fears with a startling quantity of conditional language: “We hoped that the draft circular made it clear that local health authorities were simply being invited to explore the possibility of obtaining the necessary co-operation in order to decide whether to have a local education scheme.”^26^ Apparently mollified, the GMSC relented, but responded that “it would wish to be consulted again in the light of experience of the pilot survey before giving its approval to any general scheme for cancer education.”^27^

Finally, in late August 1953, more than six years after Malcolm Donaldson had started urging all the organizations involved to take up lay cancer education, the Ministry of Health issued its circular encouraging local authorities and voluntary bodies to develop exploratory cancer-education schemes.^28^ But enthusiasm for such projects might well have been weakened by the BECC’s GP survey, finally published just a month before the Ministry’s circular. More than five thousand practitioners responded, about one response for every four questionnaires mailed: 2,148 believed that a program of lay education would be worthwhile, 2,683 thought not, and 222 qualified their yes or no answers. Campaign spokesman Lord Horder explained to *Lancet* readers that, given this result, the BECC had decided to “consider the matter further in the light of this expression of general-practitioner opinion”—in other words, to do nothing.^29^

Why did lay cancer education cause all this “anxious thought” in the immediate postwar years? Clearly, much of this caution from all concerned was a product of the delicate postwar balance between voluntary, official, and professional groups. In the contentious political stew surrounding the introduction and implementation of the National Health Service, all these organizations—the Ministry, the BECC, the BMA, even the CCHE—were attempting to shore up, reassert, or claim a voice in postwar medical policymaking. Given the volatile debates at the same time about [Other P-123] practitioner remuneration, hospital control, and research funding, it is hardly surprising that even on an apparently tangential matter such as lay cancer education the Ministry wanted neither to offend the BECC or (much worse) the BMA, nor to cede any territory to them. Meanwhile, the relatively powerless CCHE could do little without support from another organization, except to develop some pamphlets and its model scheme for others to use. The repeated emphasis on local schemes also makes sense, given the context: by encouraging local authorities to experiment with lay cancer education, the Ministry could counter the frequent charge that it was overcentralizing the nation’s health services. Furthermore, local schemes not only allowed local authorities to display a measure of independence, they shifted the costs and effort of lay education—and the possibility of failure—away from the Ministry.

But these groups’ leaders had other reasons for being anxious about lay cancer education, which drew on their assumptions about lay irrationality. Their most common objection (also common in North America) had always been that education would stir up fear rather than eliminating it; now, given the pressures on the new NHS, such fears seemed especially problematic. Claiming that the public was liable to self-diagnosis, opponents of cancer education fretted that a discussion of symptoms would drive “neurotic” Britons to surgeries, while others who might actually have cancer, now paralyzed by fear, would stay away. Worst of all, already harried GPs, overwhelmed by the crowds in their waiting rooms, would be likely to miss some early cancers while giving patients a false and perhaps disastrous sense of security.^30^ Ministry correspondence about cancer education repeatedly expressed such concerns; a 1949 memorandum to the SCRAC, for instance, closed by stating that “the number of cases coming up for diagnosis and found not to be suffering from cancer might be increased very substantially,” and asking: “Could the hospitals cope with the situation?”^31^ By the early 1950s policymakers had realized that NHS costs would be incredibly difficult to rein in. The prospect of Britons’ insisting on even more medical attention and diagnostic services, when it was unclear whether these would be certain to lower cancer mortality, must have been a frightening one. It was far better, the Ministry and the [Other P-124] BECC agreed, to teach GPs how to look for cancers *before* educational campaigns encouraged fearful Britons, newly empowered by a health service that was free at the point of delivery, to demand medical attention. The Ministry and BECC thus channeled their efforts into improving GPs’ diagnostic skills through educational materials for practitioners rather than the public.

Advocates of lay cancer education made rebutting the arguments about cancerphobia and its implications for health services a priority. First, they claimed that, when conducted in other countries, cancer education had not sowed fear. Some cited the experience of health authorities in places like Massachusetts, where cancer education had been under way for several years. Others tried to sway opponents with home-grown proof. Malcolm Donaldson, for instance, administered a questionnaire to the middle-class clubwomen who attended his lectures, and they responded that the lecture had “relieved my mind,” rather than “increased my worry.”^32^ Some educational advocates even argued that crammed waiting rooms were inevitable, as was the occasional neurotic or hypochondriac. As the team behind a Sheffield “pilot trial” of early detection insisted, “the problem is not that of cancerphobia specifically, but the vastly greater one of neurosis generally.”^33^ It was the medical profession’s responsibility, they concluded, to allay the public’s cancer fears, not dismiss them.

Equally substantial were concerns that lay cancer education might further pressure overburdened GPs, at a time when the ire of many general practitioners—whether directed at consultants, Ministry of Health administrators, or demanding patients—was palpable. Many medical professionals believed that the NHS threatened the GP’s status, for the GP no longer seemed an independent professional who owned his or her practice and conducted it according to his or her druthers. The initial levels of remuneration that the NHS provided to GPs were lower than expected, and the increase in paperwork and patient load was sizeable. The 1952 Danckwerts Award partly settled pay issues, but the nation’s GPs and the organization that claimed to speak for them, the BMA, remained concerned about workload.^34^ Understandably, then, the organizations considering cancer [Other P-125] education did not want to get on the wrong side of the nation’s general practitioners—at least, not over this subject. This also explains why the organizations concerned were careful to claim that they were considering the GP’s opinion. When advocates of cancer education set out to disprove the “crowded surgery” argument, they needed to reassure two audiences at once: reluctant GPs, and their equally reluctant leadership.

Another pervasive objection, less clearly articulated but frequently invoked, centered on the form that lay education might take. Many in the cancer elite seemed to think that lay cancer education meant an “American-style” campaign, which they insisted was unsuitable for Britain. Usually they failed to explain what they meant by “American-style” education, but it was the American Cancer Society’s mass-media campaign they disliked. BECC and Ministry of Health leaders had long been doubtful of such efforts generally, and their opposition crystallized after a delegation representing Britain’s cancer establishment toured North American research, clinical, and organizational centers in 1948. One delegate, Stanford Cade of Westminster Hospital, seemed equivocal about the ACS’s educational work, writing: “It has rendered cancer a reality to the man in the street.”^35^ Others, including Brian Windeyer of the Middlesex Hospital and F. B. Tours, the BECC’s secretary-general, were less measured. Windeyer, an internationally respected radiotherapist, objected strongly to what he saw as a constant emphasis on fear. Tours wrote almost wistfully of the great sums of money the ACS had raised, but then criticized the “glaring” posters and advertisements that constantly reminded Americans of cancer’s death toll.^36^ At the tour’s end, Cade, Windeyer, Tours, and their leader, Lord Horder, agreed that what they saw as a “strident” appeal to fear, while it might succeed in the United States, was wrong for Britain. Cementing their rejection of the American approach was a statistic that they found especially alarming: cancer-detection clinics in the United States had waiting lists of up to eight months, because Americans had taken the gospel of early detection to heart.^37^ The ACS might have viewed this fact as an unfortunate but temporary sequela of success, but the British visitors took it as an indictment of not only the approach, but also its execution.[Other P-126]

These objections to the ACS’s approach—that its appeal to fear would not work on the other side of the Atlantic, and that it might cause even more problems than it solved—featured in almost all discussions of cancer education over the next decade. Though they never articulated what they thought constituted the British national “character,” the nation’s elite clinicians and health officials maintained that using fear to spur Britons to action was unsuitable.^38^ Even the enthusiastic Donaldson admitted that “in America it is put across in the wrong way.”^39^ Neither the opponents nor the proponents of lay cancer education had hard evidence regarding whether the high-visibility, hard-sell approach they identified with American campaigns would succeed or fail in the British context. Certainly they made no reference to formal, structured assessments of the British mind-set, such as Mass Observation or other social surveys. Rather, they—like many of those engaged in health education in the first half of the twentieth century, on both sides of the Atlantic—relied on assumptions about the nature of a public they thought they knew well enough. Given these objections, if cancer education were to be accepted as valid, it would have to prove its merit in terms that made sense to the cancer elite.

This attitude toward education illustrates a key difference between the British cancer elite and the Americans whose work they observed and analyzed: American cancer workers seem to have decided fairly early on that education would cut delay, which would reduce cancer mortality, and then to have gotten on with it—from the early campaigns of the 1920s, through the increased activity of the late 1930s, to the full-scale media onslaught of the late 1940s. Robert Aronowitz suggests that the delay argument had a “self-evident” rationale, and its American advocates were relatively unconcerned with collecting “robust data” to support the argument.^40^ Indeed, it was well after these educational initiatives began that some American investigators collected statistical evidence intended to prove education’s value, suggesting that an apparently improving stage distribution and growing overall presentation rates were at least partly a result of educational activities.^41^ By contrast, the British cancer elite demanded that proof *before* they would start a broad cancer-education campaign, and even then they remained skeptical: even the most positive assessments [Other P-127] of American cancer campaigns, for instance, still concluded that health education’s effects were “imponderable” and nearly impossible to prove statistically.^42^ In the end, finding proof that such campaigns could work in Britain would fall to the organizers of an “experiment” offering a very different approach to both education and the public.

## Rethinking Knowledge, Fear, and Ignorance: The Manchester Educational Experiment

The Manchester Committee on Cancer (MCC) began its educational experiment in the early 1950s, having done independent fund-raising for cancer research and other publicity activities in the northwest since the 1920s.^43^ The interest in cancer education in this group was led by Ralston Paterson, the radiotherapist director of the Christie Hospital and Holt Radium Institute. Admired for his organizational skills, Paterson designed some of the first randomized controlled trials in cancer therapy. He also stressed the importance of statistical investigation as a tool for assessing practice, an orientation that informed his approach to cancer education. His team included Jean Aitken-Swan, a Christie social worker, and John Wakefield, the MCC’s executive officer.

The Manchester team’s “experiment” simultaneously drew on some common assumptions about cancer education and tested others. The group began with the proposition that the problem to be solved was delay, especially women’s delay in seeking medical attention for what would turn out to be breast or cervical cancers. These experts also presumed that while the object of a cancer-education campaign was to dispel fear, education itself, if incorrectly undertaken, might *spread* fear instead. As a result, the Manchester group described much of its plan in terms of what it would *not* do. Their work would not be negative, they claimed, and they would especially avoid what they termed “the lurid ‘This might happen to you’ approach.”^44^ Nor would they stress “cancer signs,” as was common [Other P-128] in North American cancer education; instead, potential symptoms were described as “abnormal” and thus as a reason to consult a doctor. The group would not oversell its message by making broad claims like “cancer is curable,” or even “early cancer is curable.” Finally, the Manchester experiment would not take what MCC leaders called “the grand approach with large audiences and spectacular publicity,” but would proceed through informal talks to small groups, short items in local newspapers, and short pamphlets.^45^

Especially distinctive were the steps that Paterson’s group took to devise this experiment so that its results would be as statistically convincing as possible. Before launching the campaign, the MCC commissioned large social surveys of women in Manchester city, Salford, and Stockport, while Christie personnel conducted in-depth interviews with cancer patients and their family members.^46^ These surveys promised a baseline assessment of the population, allowing the group to have a measurable sense of what exactly had changed in the meantime, presumably at least partly due to the educational program. While others involved with cancer education used opinion polls to judge the state of public knowledge, they tended to rely on national surveys rather than local ones, and it is unclear whether they used the surveys to measure the effects of particular programs.^47^ The Manchester group, by contrast, expected surveys and interviews to explain “why women put off seeking advice for symptoms which they suspect to be cancer.”^48^

As expected, the questionnaire demonstrated that women of all social classes considered cancer the most alarming disease. But what was both troubling and illuminating, the Manchester team decided, was that a substantial proportion of the women surveyed (from half to three-quarters) believed that cancer was *never* curable.^49^ This contrasted with the two-thirds [Other P-129] of Americans surveyed who said that cancer could be cured.^50^ Further questioning turned up another important finding: for some cancers, the problem was not one of ignorance. Only 40% of those surveyed thought that “a show of blood or discharge ten years or so after the change of life” might mean cancer. However, nearly 40% ranked a painless lump in the breast as the most alarming of a number of symptoms (including “constant cough” and “losing weight”), and 83% of those said that their alarm was because it was a sign of cancer.^51^ Most revealing, though, were the responses from the open-ended questions included in the survey. Even among women in the “average +” group who held “presumably the most enlightened opinion,” the pain and apparent incurability that they associated with cancer worried them the most:

“The dreadful pain that one automatically associates with cancer.”

“The pain is so terrifying.”

“The suffering is so intense and usually so long drawn out.”

“It always seems to prove fatal.”

“The widespread idea that they can’t do anything about it.”^52^

This pessimism, the Manchester group agreed, was the chief problem that lay education needed to address.

Detailed interviews conducted with cancer patients and their families seemed to support these conclusions. Social worker Jean Aitken-Swan interviewed 239 women and 75 men who were Christie patients or their relatives.^53^ The results, Aitken-Swan and Paterson argued, demonstrated that two distinct problems accounted for delay, for the patients interviewed (or described by relatives) fell into two distinct groups, with different behavior patterns regarding symptoms and medical advice. Some patients were judged to be genuinely ignorant: they did not know what their symptoms might mean. “Not-knowing,” as Aitken-Swan and Paterson put it, accounted for their failure to suspect cancer and thus consult medical professionals. Sometimes this was because the symptoms were less commonly associated with cancer, or seemed related to recent pregnancies or previous health problems. This explained most delay in cases [Other P-130] of cervical, skin, and mouth cancer.^54^ By contrast, many patients “knew” perfectly well that their symptoms might mean they had cancer. Some who “knew” sought medical attention quickly, especially younger patients who seemed more inclined generally to consult medical professionals. But a significant remainder of patients “knew” but delayed more than three months, sometimes more than a year; these patients, then, formed “the main challenge to any public education project.”^55^ Many—especially breast cancer patients—presented themselves for diagnosis and treatment only when their symptoms became too problematic to manage or when relatives forced them to.

What, then, accounted for why some people who “knew” chose to act and others chose to delay? Differences in intelligence did not explain it, as IQ testing showed. The answer, Aitken-Swan and Paterson suggested, was fear—and the defensive psychological reactions that sometimes arose from it, such as denial, suppression and rationalization, and fatalistic acceptance. But the fear was not always of the cancer per se, but of what these patients associated with it: invalidism, hospitals, doctors, treatment, and, especially among older patients, anxiety about dependency, “of ‘being sent away’ and losing their rooms or houses. So much depended on their keeping going, and they saw no hopeful outcome if they let themselves get into the hands of the hospitals.”^56^ Interview excerpts quoted by Aitken-Swan and Paterson illustrated this:

One timid, elderly lady said, “That’s the God’s truth, and I wouldn’t tell you a lie. It was just that I was frightened I’d be sent away. I couldn’t help it.”^57^

The Manchester group believed that these reactions were problematic, sometimes citing others who claimed that delay was evidence of psychological pathology.^58^ Nevertheless, their publications maintained [Other P-131] a relatively sympathetic tone. For instance, in 1957 John Wakefield told prospective educators:

Fear of cancer seems to dwell on a deep emotional level; and any scheme to alleviate fear must therefore try to influence this same primitive level of emotion. People must be convinced that they and their families will be better off if they act in the way we urge them. This is no easy task. For almost all the evidence before them confirms people in their belief that cancer is a distressing, painful and inevitably fatal disease for which doctors can do little.^59^

While he referred to the fear of cancer as a “primitive” emotion, Wakefield here, as always, reminded medical professionals and other educators that everyday people had rational reasons for dreading cancer. He also insisted that

the divergent paths of learning of speaker and audience have created a gap that [the speaker] must try to bridge. He may think his audience of cotton operatives very ignorant of elementary matters of medicine; but to them he will seem no less of an ignoramus on the intricacies of cotton spinning.^60^

Such statements highlight a difference between this approach and the usual rhetoric deployed by North American and British advocates of cancer education: the latter portrayed the audience for instruction as an ignorant, irrational mass in need of expert guidance; while the Manchester group described an audience of unschooled but not necessarily unintelligent everyday people who acted reasonably, given the evidence available to them.

This perception was at the heart of the Manchester group’s assessment of what was at stake in cancer education. The problem facing them was not public ignorance of cancer symptoms, but the public’s knowledge of the disease’s all-too-frequent consequences. As Paterson had argued, “One could almost say axiomatically that every adult woman knows what a *lump* in the breast *may* mean.”^61^ Rather, the problem was the widespread fear of what would happen if one had cancer: pain, suffering, dependency, and death. And, the Manchester group pointed out, the public’s pessimism about cancer had a “very substantial justification,” for “over *all* the cancers taken together, death is still a much more common outcome than cure, and the manner of death is, by and large, often as distressing as can be.”^62^ Aitken-Swan’s interviews with families of deceased cancer [Other P-132] patients showed how many patients in the terminal stages of cancer were unable to obtain admission to the hospital where they had initially been treated, or to any hospital at all. Some patients had attentive GPs who visited regularly, and their families were usually satisfied with the care the patient had received—but other families argued that their GP had lost interest in the patient once it was clear that the patient was dying. The family members knew very well what the terminal patient experienced, as in many cases they had borne the chief responsibility for nursing.^63^ “This,” Aitken-Swan and Paterson wrote, “is cancer as the general population knows it—unpleasant, incurable, and rejected.”^64^ So pervasive was this experiential knowledge that it had “a direct bearing on the public’s lack of confidence in what can be done for the cancer patient,” and “lack of confidence in the efficacy of any treatment” accounted for delay.^65^

Paterson’s group argued that their approach to lay education—a quiet but incessant barrage delivered through already-established community networks—was the best weapon against such a “climate of fear.”^66^ They chose three boroughs—Bury, Rochdale, and Oldham—surrounding Manchester city proper, with a largely working-class population of 620,000. After explaining the plan to hospital staff, BMA branches, and medical officers of health, Wakefield contacted local organizations—civic groups, women’s clubs, church groups, political party branches, cooperative guilds, pensioners’ associations, and even sports clubs—to schedule talks during routine meetings. He also visited the editors of the twelve local newspapers that covered the target area, to “ensure the accurate reporting of meetings and to avoid unfortunate sub-editing.”^67^ Speakers were drawn largely from the Christie Hospital’s staff, although Wakefield himself frequently filled in. Meanwhile, the local newspapers reported [Other P-133] on the talks given at local meetings, aided by a condensed version of the lecture mailed to them afterward. And as a follow-up to the lectures, the MCC mailed copies of two pamphlets (on breast and on cervical cancer) to each group’s secretary about two weeks after the talk was given. Although Wakefield later admitted that this approach did not directly reach “the apathetic and the unclubbable,” it did, he argued, reach into the heart of the community, creating “a considerable ‘scatter-effect,’ an outward-spreading ripple of enlightenment . . . potentially one of the most powerful of all [influences] in changing existing attitudes.”^68^

This emphasis on low-key, everyday efforts to change community attitudes and beliefs from within even produced a new form of cancer education: the voices of former sufferers themselves. After observing the “dramatic” impact on a group when a member testified how she had been cured of cancer, Wakefield and his colleagues took to tape-recording the stories of everyday residents of the community and playing them at other meetings. Here Wakefield invoked one of the few social analysis texts that would guide the Manchester group’s educational experiment, or at least the only one the group ever cited: Richard Hoggart’s chapter on “Them” and “Us” in *The Uses of Literacy: Aspects of Working-Class Life*.^69^ Allowing an everyday person to take the stage, Wakefield insisted, was remarkably effective, for “her words were accepted without any of the reserve accorded to the claims of someone outside the group, the unvoiced and perhaps unconscious suspicion that they are being ‘told the tale.’”^70^ Cured-cancer clubs in the United States were intended to attract media attention; by contrast, the Manchester group used recognizably local voices to tell convincing stories about cancer treatment directly to the public.

Did it work? By the late 1950s, the Manchester group argued in print that their educational interventions had succeeded, albeit in a limited way. To assess progress, they examined changes in public opinion in their target area, as measured against public opinion in a “control” area (Preston, Blackburn, and Wigan); analyzed trends in delay compared to those in control communities; and conducted interviews to see whether the campaign had changed individual patients’ behavior. The surveys returned mixed results, although the team tried to portray them in a generally positive light. When asked in 1957 if cancer was curable, a much greater proportion of women surveyed in the experimental area responded that it was (55%), compared to women from the control area surveyed at the same time (46%) and [Other P-134] women from the area adjacent to the experimental zone four years earlier (36%).^71^ Furthermore, a greater proportion of women surveyed in the target area now seemed to believe that early treatment increased the chances of a cure (71%). These results were statistically significant, and seemed convincing that the educational program had produced, “though in modest degree only, a real change in public opinion.”^72^

Nevertheless, other measures suggested that the experiment’s success had been limited. For instance, a slightly greater proportion of women in the control area correctly identified the “first signs” of cervical and breast cancer than did women in the experimental area. The skeptic, Paterson’s team admitted, could interpret this as “implying that in an area where there has been five years of cancer education there is rather less knowledge about the symptoms than in the control area.”^73^ Not surprisingly, the Manchester group took a more positive approach, arguing that the goal had *not* been to teach women to identify cancer symptoms; rather, they had stressed significant symptoms only when they could have “linked this with the emphasis that ‘these do not necessarily mean cancer by any means—they merely mean that you should see your doctor and be examined.’”^74^ The group also put a positive spin on the fact that only 8% of women surveyed in the experimental area had heard of talks on cancer being given: after all, they pointed out, only 11% actually belonged to a women’s group, so by their (questionable) reasoning, eight of every eleven members of such groups had been reached. Rather than using this finding to indict their choice of campaign focus, the Manchester group concluded that they simply needed to expand their efforts, perhaps through factory lectures.^75^

But while women’s opinions had apparently shown some evidence of change, the important question remained: had women with this “improved outlook on the curability of some cancers and on the value of early treatment” acted on this?^76^ Here the Manchester team brought out their own statistics drawn from the Christie Hospital, where, they pointed out, “the great majority of [Manchester-area] patients with breast and cervix cancer . . . are sooner or later referred.”^77^ There did seem to be [Other P-135] a small but steady improvement in the numbers of cancer patients who had sought medical attention for their symptoms less than a month after noticing them, from 28% in 1950–51 to 38% in 1954 –55. This improvement was not quite, but “very nearly” statistically significant, and no such improvement was visible in the control area.^78^ Furthermore, the proportion of women with breast and cervical cancer who “presented” while in stages I and II seemed to have risen, from 55% to 64% of breast cancer patients and from 50% to 70% of cervical cancer patients. Interviews with patients likewise turned up equally suggestive but not conclusive results. A relatively large number of patients from the experimental area recalled having heard about the campaign’s talks (28%) or having read articles in the local papers about these talks (35%). And many of the women who “took immediate action” in response to a symptom of breast cancer—in other words, who consulted a physician within a month—cited the campaign as the main factor motivating them. In fact, 36 of the 211 breast-cancer patients in the “immediate action” group attributed their action to the campaign.^79^

In the end, Paterson and his team claimed limited success for their educational campaign. They argued that there had been “a change in opinion and action,” especially for breast cancer, even though that change was “extremely gradual” and “effected through a minority of the women reached.”^80^ This, they admitted, could be attributed to “an overall improved attitude to ‘doctoring’ and an increased willingness to seek advice for all abnormal or disturbing symptoms.”^81^ But the team also felt that, given their own “stringent” measures of success, they might have to continue education for a longer time in order to produce substantial results, and they indicated that they might consider a slight change in tactics: while talks had apparently been more influential than newspaper articles in shaping behavior, they clearly did not reach as large an audience as expected. (Not long after the initial experiment was finished, the MCC sent a “Mobile Information Unit” staffed with nurses to visit offices, stores, and factories.) Finally, the group hit on a more interesting conclusion: they might accept that the number of women whose attitudes and behavior would be affected by an educational campaign would be small. However, Aitken-Swan and Paterson concluded, perhaps this minority could be convinced to influence their fearful sisters, since “the advice and [Other P-136] persuasion of friends” was said to be the second most important factor in convincing women with symptoms to seek medical advice.^82^

## Conclusion

The Manchester approach to lay cancer education satisfied many critics among the British cancer elite that education could be valuable, and Paterson and Wakefield soon became de facto experts on the subject, frequently consulted by the Ministry of Health and others. (Wakefield even became chair of the International Union Against Cancer’s Educational Committee.) Cancer education itself, however, remained a relatively low priority for the Ministry and other groups until the 1960s. As Virginia Berridge and Kelly Loughlin show, it was not until the link between smoking and lung cancer was established that British health organizations embraced national advertising-style cancer-education campaigns.^83^ Meanwhile, the MCC continued experimenting with cancer education locally, in hopes of demonstrating its value nationally. In the 1960s and 1970s, they and a similar group in nearby Merseyside produced more social-scientific evaluations of women’s knowledge and practices regarding cancer, using these analyses to judge the efficacy of local programs. Together with the published reports of the Manchester group’s 1950s work, these are good sources for documenting everyday British women’s ideas about cancer and its treatment. Most popular media of the period seem to have discussed cancer in terms of high-tech research accomplishments and gleaming new facilities. By contrast, surveys and interviews such as the Manchester group’s hint at how cancer (and chronic and terminal diseases generally) fit into the social landscape of everyday Britons.

The discourse about public cancer education offers us insight into the concerns of the cancer elite about postwar medical care. We have seen how discussions about an “irrational” public stood in for concerns about how, or even whether, the public could be convinced to use health services wisely—that is, according to the goals set out by medical professionals and health planners. Much of the postwar British cancer elite shared with North American counterparts what we might call a “deficit” model of education, assuming that the problem facing cancer authorities was the ignorance and irrationality of the everyday Briton. But knowledge, they feared, would in this special case simply exacerbate public fears, by encouraging worried women and men to seek attention from a system [Other P-137] that was organizationally unprepared for them and already struggling to control costs. In such a climate, it was perhaps inevitable that the cancer elite would demand evidence that lay education could be made to work in Britain before they would risk carefully negotiated organizational relationships and restricted resources.

By contrast, the Manchester group’s reconceptualization of lay cancer education and the problems it was meant to solve was surprisingly well suited to the contentious medical economy of postwar Britain. By avoiding discussions of specific symptoms, it released proponents of lay cancer education from the charge that they encouraged self-diagnosis; furthermore, it argued (optimistically) that the public could be taught to use health services in a rational way. Indeed, perhaps the most interesting aspect of the Manchester team’s approach to cancer education was that it redistributed the burden of action among the public to be educated, the educators who spoke to them, and even the experts responsible for organizing cancer services. After all, if educators hoped to sell the good news about cancer to the public, they needed cancer centers, hospitals, and specialists to do the good work that would make the positive results of “modern” treatment visible. In a world where cancer sufferers still all too frequently died painful and lonely deaths, the Manchester group admitted that it would be slow and difficult to change the average experience of cancer, and thus the average person’s understanding of it. Although the educational experts remained in charge of that effort, what made the Manchester experiment unique was the degree to which its leaders were willing to look to everyday Britons—if only temporarily—to understand their version of “the truth about cancer.”[Other P-138]

## Footnotes

1
	  R. Gedling to R. Brain and W. P. Kennedy, September 1949, MH 55/927, National Archives, Kew Gardens, Surrey, U.K. (hereafter NA).
	

2
	  On American cancer education, see PattersonJames T.The Dread Disease: Cancer and Modern American CultureCambridgeHarvard University Press1987 ReaganLeslie J.“Engendering the Dread Disease: Women, Men, and Cancer,”Amer. J. Pub. Health199787177987936663510.2105/ajph.87.11.1779PMC1381161 LernerBarron H.The Breast Cancer Wars: Hope, Fear, and the Pursuit of a Cure in Twentieth-Century AmericaNew YorkOxford University Press200011949674 AronowitzRobert“‘Do Not Delay’: Breast Cancer and Time, 1900–1970,”Milbank Quart.2001793558610.1111/1468-0009.00212PMC275120211565161 GardnerKirsten E.Early Detection: Women, Cancer, and Awareness Campaigns in the Twentieth-Century United StatesChapel HillUniversity of North Carolina Press2006 . A brief comparison of American and British programs is PattersonJames T.“Cancer, Cancerphobia, and Culture: Reflections on Attitudes in the United States and Great Britain,”Twent. Cent. Brit. His.199121374910.1093/tcbh/2.2.13711622988 . On cancer and disease education in twentieth-century Britain, see CantorDavid“Representing ‘the Public’: Medicine, Charity and Emotion in Twentieth-Century Britain,”Medicine, Health and the Public Sphere in Britain, 1600–2000SturdySteveLondonRoutledge200214468 BerridgeVirginiaLoughlinKelly“Smoking and the New Health Education in Britain, 1950s–1970s,”Amer. J. Pub. Health200595956641591481610.2105/AJPH.2004.037887PMC1449291 ; and Ornella Moscucci’s unpublished “Fast-Track to Treatment: Cancer Education in Britain, circa 1900–1948,” distributed for Patients and Pathways: Cancer Therapies in Historical and Sociological PerspectiveUniversity of Manchester102005
	

3
	  Cantor, “Representing ‘the Public’” (n. 2).
	

4
	  Moscucci, “Fast-Track to Treatment” (n. 2), pp. 9–10.
	

5
	  Reagan, “Engendering” (n. 2); Gardner, *Early Detection* (n. 2), esp. chap. 1.
	

6
	  Aronowitz, “‘Do Not Delay’” (n. 2); Lerner, *Breast Cancer Wars* (n. 2), pp. 41–60.
	

7
	  For instance, RavenRonald W.Gough-ThomasJoan“Cancer Education of the Public,”Lancet195125849596 on p. 496 10.1016/s0140-6736(51)91751-514874421
	

8
	  Moscucci, “Fast-Track to Treatment” (n. 2), p. 8.
	

9
	  CantorDavid“The Definition of Radiobiology: The Medical Research Council’s Support for Research into the Biological Effects of Radiation in Britain, 1919–1939” Ph.D. diss. University of Lancaster1987
	  
	

10
	  “Notes for Lord Nathan,” 14 September 1954, in SA/CRC/Q.1/3, Papers of the Cancer Research Campaign, formerly the British Empire Cancer Campaign (hereafter BECC Papers), Special Collections, the Wellcome Library for the History and Understanding of Medicine, London (hereafter Wellcome Library).
	

11
	  Practical obstacles also intervened, such as wartime paper restrictions.
	

12
	  For instance, F. L. Hopwood to F. B. Tours, 24 September 1952, SA/CRC/Q.1/2, BECC Papers.
	

13
	  “Notes” (n. 10), BECC Papers.
	

14
	  Ibid.
	

15
	  F. B. Tours, January 1953, SA/CRC/R.1/4, BECC Papers.
	

16
	  Executive Committee Minute 1915, SA/CRC/Q.1/5, BECC Papers.
	

17
	  R. Brain, [August 1949]; Gedling to Brain and Kennedy (n. 1); and M. Reed, “Cancer Propaganda,” 21 July 1947; all NA MH 55/927. This was part of a larger debate about the CCHE’s functions relative to the Ministry of Health. In 1946–47, the Ministry and the Central Office of Information had commissioned, circulated, and revised a script for a lay cancer film, but chose to concentrate on a film for GPs: NA MH 55/910.
	

18
	  “Note for file,” 13 September 1948, NA MH 55/927.
	

19
	  “Cancer Education: Memorandum by the Ministry of Health,” October 1949, ibid.
	

20
	  R. Gedling, 9 May 1950, ibid.
	

21
	  See the minute by M. R. [probably M. Reed], 19 July 1950, ibid.
	

22
	  A. Bavin to R. Sutherland, 9 February 1951, ibid.
	

23
	  Standing Cancer and Radiotherapy Advisory Committee, “Minutes of Meeting held 26 June 1952,” ibid.; “Central Health Services Council,”Lancet195326229513085751
	

24
	  “Note of meeting,” 13 March 1953, NA MH 55/927; “Note of meeting between Mr Malcolm Donaldson and officers of the Department on September 18, 1952,” ibid. These memos also discussed the Marie Curie Memorial Foundation’s small program of lay cancer education, conducted without much consultation with other organizations.
	

25
	  General Medical Services Committee, “Minutes of the May 12, 1953 meeting,” p. 16, *GMSC 1952–53*, part 2, British Medical Association Archives, London (hereafter BMAA).
	

26
	  Ministry of Health to GMSC, quoted in “Agenda for the July 23, 1953 meeting,” p. 33, *GMSC 1953–54*, part 1, BMAA.
	

27
	  Ibid., p. 39.
	

28
	  Ministry of Health, Circular 18/53.
	

29
	  HorderLord“The General Practitioner and Lay Education in Cancer” (letter to the editor)Lancet1953262137
	  
	

30
	  SCRAC members also pointed out that bed shortages remained acute and waiting lists long: “Minutes of Meeting held 26 June 1952,” NA MH 55/927.
	

31
	  “Cancer Education: Memorandum by the Ministry of Health” (draft, October 1949), ibid. See also Gedling to Brain and Kennedy (n. 1): “From the Minister’s point of view the problem resolves itself largely into whether, if some propaganda of the kind suggested by Dr. Donaldson is done, there will be diagnostic and treatment facilities available for those who hearken to it.”
	

32
	  DonaldsonMalcolm“Lay Education in Cancer” (letter to the editor)Lancet1953262199
	  
	

33
	  WalterJ.AtkinsonE. C.“Early Cancer Detection and Education: A Pilot Trial,”Brit. Med. J.1955162730 on p. 630 1323057410.1136/bmj.1.4914.627PMC2061392 . See also “Educating the Layman: Hunterian Society’s Debate,”Lancet1948252820
	

34
	  WebsterCharlesThe National Health Service: A Political HistoryOxfordOxford University Press1998 MorrellDavid“Introduction and Overview,” pp. 1–19, and Charles Webster, “The Politics of General Practice,” pp. 2044General Practice under the National Health Service, 1948–1997LoudonIrvineHorderJohnWebsterCharlesLondonClarendon Press1998 Grey-TurnerElstonSutherlandF. M.History of the British Medical Association21932–1981LondonBritish Medical Association1982 chap. 5
	  
	

35
	Stanford Cade, in “Report of a Visit to Canada and the United States by a Delegation from the British Empire Cancer Campaign, July 1948,” [issued 25 March 1949], p. 13, PP/FGS/E.30, F. G. Spears Papers, Wellcome Library.

36
	F. B. Tours, ibid., pp. 102–3.

37
	Ibid., pp. 13 (Cade), 41 (Windeyer), and 102–3 (Tours).

38
	LernerBreast Cancer Wars26467
	

39
	“Report on the Special Meeting of the Chelsea Cancer Committee,” 12 January 1950, p. 8, NA MH 55/927.

40
	Aronowitz, “‘Do Not Delay’” (n. 2), p. 358.

41
	RobbinsGuy F.“The Significance of Early Treatment of Breast Cancer: Changes Correlated with the Cancer Education Programs of 1940–1955,”Cancer195912688921366301010.1002/1097-0142(195907/08)12:4<688::aid-cncr2820120410>3.0.co;2-7 Aronowitz“‘Do Not Delay’” 2 380
	

42
	“Cancer Education and Earlier Diagnosis,” [probably 1953], NA MH 55/927. See also Richard Doll to Neville Goodman, 13 August 1953, ibid.; R. W. Scarff to F. L. Hopwood, 16 March 1954, SA/CRC/Q.1/3, and correspondence regarding H. H. Bentall, “Cancer Education,” January 1956, SA/CRC/Q.1/4, BECC Papers.

43
	MagnelloEileenA Centenary History of the Christie Hospital ManchesterManchesterChristie Hospital20014647
	

44
	PatersonRalstonMetcalfe BrownC.WakefieldJohn“An Experiment in Cancer Education,”Brit. Med. J.19541121921 on p. 1219 10.1136/bmj.2.4898.1219PMC208012113209087 WakefieldJohn“Cancer Education in Practice,”Education of the Public Regarding Cancer: Report of Conference Held at B.M.A. House, 24 January 1957Central Council on Health Education19572935 on p. 30 , NA ED [Ministry of Education] 50/693.

45
	Paterson, Brown, and Wakefield, “Experiment” (n. 44), p. 1219.

46
	This was actually two surveys: one of 1,200 women, specifically about cancer, and another of 1,200 women questioned at the end of a mass radiography survey conducted by commercial canvassers and health visitors. See PatersonRalstonAitken-SwanJean“Public Opinion on Cancer: A Survey among Women in the Manchester Area,”Lancet195426785761 on p. 857 10.1016/s0140-6736(54)91952-213213058

47
	By the mid-1950s the Manchester group and the Canadian Cancer Society used the same questionnaire to facilitate international comparisons: PatersonRalston“Why Do Cancer Patients Delay?”Can. Med. Assoc. J.19557393140PMC182636013276881

48
	Paterson and Aitken-Swan, “Public Opinion” (n. 46), p. 857.

49
	Fifty percent of the women responding to the cancer survey said they believed cancer could never be cured, while women responding to the same question at the end of the tuberculosis survey were more pessimistic: 55% of those asked by health visitors said cancer could never be cured, while 74% of those asked by commercial canvassers said so. The obvious conclusion, though the Manchester team did not draw it, was that women felt more comfortable admitting this pessimism to commercial canvassers than to potentially judgmental health visitors.

50
	Paterson and Aitken-Swan, “Public Opinion” (n. 46), p. 858.

51
	Ibid., p. 859.

52
	Ibid., p. 860.

53
	These interviews seem not to have survived.

54
	Aitken-SwanJeanPatersonRalston“The Cancer Patient: Delay in Seeking Advice,”Brit. Med. J.1955262327623241323057310.1136/bmj.1.4914.623PMC2061366
	

55
	Ibid., pp. 624–25.

56
	Ibid., p. 625. This resembles the attitude found by Lucinda McCray Beier’s studies of health in working-class Lancashire communities: Beier“Expertise and Control: Childbearing in Three Twentieth-Century Working-Class Lancashire Communities,”Bull. Hist. Med.2004783794091521105310.1353/bhm.2004.0056 Beier“‘We Were Green as Grass’: Learning about Sex and Reproduction in Three Working-Class Lancashire Communities,”Soc. Hist. Med.20031646180

57
	Paterson, “Why Delay?” (n. 47), pp. 933–34.

58
	See, e.g., HendersonJ. G.WittkowerE. D.LougheedM. N.“A Psychiatric Investigation of the Delay Factor in Patient to Doctor Presentation in Cancer,”J. Psychosom. Med.19583274110.1016/0022-3999(58)90014-x13570633 , cited by WakefieldJohn“A Co-operative Scheme of Public Education about Cancer,”Brit. J. Clin. Pract.1961151656713782596

59
	Wakefield, “Cancer Education in Practice” (n. 44), p. 32.

60
	WakefieldJohnCancer and Public EducationLondonPitman Medical Publishing196226
	

61
	Paterson, “Why Delay?” (n. 47), p. 934 (emphasis in original).

62
	Ibid., p. 935.

63
	Aitken-SwanJean“Nursing the Late Cancer Patient at Home: The Family’s Impressions,”Practitioner195918364 6913792206
	

64
	Aitken-Swan and Paterson, “Cancer Patient” (n. 54), p. 627. This reiterated what a 1952 survey found: a substantial proportion of dying cancer patients were dependent on family, friends, and neighbors for care, and many were aware that they had been “sent home from hospital to die” ( Joint National Cancer Survey Committee of the Marie Curie Memorial and the Queen’s Institute of District Nursing, *A Report on a National Survey Concerning Patients with Cancer Nursed at Home* [Marie Curie Memorial, April 1952], p. 25, SA/QNI/P.1/5, The Queen’s Nursing Institute Collection, Wellcome Library). See also Noémi Tousignant, “Exposing Relief: Place, Cancer Pain and Appropriate Care for the Dying in Britain, 1950–1980” (M.Sc. thesis, University of Manchester, 2001).

65
	Aitken-Swan and Paterson, “Cancer Patient” (n. 54), p. 627.

66
	Wakefield, “Co-operative Scheme” (n. 58), p. 166.

67
	Paterson, Brown, and Wakefield, “Experiment” (n. 44), pp. 1219–21, on p. 1220; Paterson, “Why Delay?” (n. 47), p. 936.

68
	WakefieldCancer and Public Education 602324
	

69
	HoggartRichardThe Uses of Literacy: Aspects of Working-Class LifeLondonChatto and Windus1957
	

70
	WakefieldCancer and Public Education6027
	

71
	PatersonRalstonAitken-SwanJean“Public Opinion on Cancer: Changes Following Five Years of Cancer Education,”Lancet195827279193 on p. 791 1358900710.1016/s0140-6736(58)91494-6
	

72
	Ibid., p. 792.

73
	Ibid.

74
	Ibid., pp. 792–93

75
	Ibid.

76
	Ibid., p. 793.

77
	Aitken-SwanJeanPatersonRalston“Assessment of the Results of Five Years of Cancer Education,”Brit. Med. J.1959170812 on p. 709 1362910610.1136/bmj.1.5123.708PMC1993086
	

78
	Ibid., p. 709.

79
	Ibid., p. 711.

80
	Ibid.

81
	Ibid., p. 712.

82
	Ibid.

83
	Berridge and Loughlin, “Smoking” (n. 2).

